# Impact of urban facilities spatial inequality on sustainable travel mode

**DOI:** 10.1371/journal.pone.0308610

**Published:** 2024-10-31

**Authors:** Jorge Urrutia-Mosquera, Luz Flórez-Calderón, Yasna Cortés, Rodrigo Troncoso, Marcelo Lufin

**Affiliations:** 1 Department of Economics and Institute for Applied Regional Economics (IDEAR), Universidad Católica del Norte, Antofagasta, Chile; 2 Industrial Engineering Department, Universidad Católica del Norte, Antofagasta, Chile; 3 Faculty of Government, Universidad del Desarrollo, Santiago, Chile; King Abdulaziz University, SAUDI ARABIA

## Abstract

With the implementation of sustainable development objectives in developing countries, urban planning, land use regulation, and urban mobility policies are expected to help reduce inequalities in access to urban facilities. Urban transport policies are also expected to encourage travel by non-motorised modes and public transport. These are considered to be the sustainable modes of urban transport. In this paper, we investigate how inequality of urban facilities impacts trips made by sustainable modes in the city of Santiago de Chile. We use a Poisson regression model and its geographical extension, the geographically weighted Poisson regression model (GWPR). The results suggest that the inequality of urban facilities impacts trips made by sustainable modes. The variables with the highest relevance are the spatial distribution of mixed land use, the spatial distribution of urban services related to transport infrastructure, primary and secondary education, as well as the spatial distribution of demographic variables related to people’s life cycle.

## Introduction

Promoting sustainable mobility in all its dimensions (increased public transport travel, increased non-motorised travel, and the promotion of electro-mobility) has been considered the alternative solution to reduce the CO2 emissions the transport sector produces. This sector is the second largest source of emissions in the world, accounting for between 23% and 25% of total global CO2 emissions of all sectors [1–3]. In Chile, as in the rest of the world, the transport sector represents the second largest source of CO2 emissions. Data from the Ministry of Energy show that in 2016, the transport sector contributed 26.94% to the total CO2 emissions produced in the country.

Moving towards sustainable mobility models is important because increasing the use of sustainable modes of transportation can mean a future emissions reduction of 1.3 tonnes of CO2 per year [4]. However, achieving this goal requires a strong urban development component and public policies oriented toward sustainable mobility [1, 5].

Studies on spatial inequality and its impacts on urban transport use in different cities worldwide provide evidence that the unequal distribution of urban facilities in space is related to the urban mobility patterns of people, particularly the modes of transport used [6]. There is evidence that limitations in urban development in cities accompanied by the absence or limited existence of urban transport infrastructure generate negative effects on the most vulnerable segments of the population [7–9], e.g., exposure to long travel times and long distances for subsistence and personal care activities [10–12].

Urban, social, and economic inequalities in cities are, to some degree, a product of how cities are designed, developed, and grown [13–15] since spatial mobility and the intensity of population movements depend on a large extent on the characteristics of urban contexts and the socioeconomic profiles of residents. For example, [16, 17] suggest that mixed land uses, well-connected streets, and retail activities closer to residences induce non-motorised transport and reduce reliance on private car use. There is also evidence that if private vehicle ownership is affected and public transport travel options are improved, public transport becomes more competitive and attractive [18]. The experience of some cities in developed countries, which prioritise sustainable mobility based on public transport and non-motorised travel, is characterised by building neighbourhoods with access to a range of amenities that benefit walking and cycling [19, 20].

The promise of sustainable urban development seeks to reduce spatial inequity in the distribution and provision of goods and services in cities [21], which is why the conception of the city, the way goods and services are distributed in space, and the configuration of land use determine the mobility dynamics and travel behaviour of its residents [22, 23].

Urban and transport planning at different scales is expected to benefit sustainable mobility [24] and contribute to thinking and designing public spaces, where mobility is a function of pedestrians, public transport, and not private vehicles [25]. In this sense, some empirical work [11, 26] suggests that intra-urban and inter-urban differences related to access to goods and services and urban amenities, especially those related to transport infrastructure, have significant impacts on travel behaviour.

Therefore, the new mobility perspective seeks to reduce some of the adverse effects, such as congestion [27], air pollution [28, 29], and increased travel times, derived from the car-based transport model [30]. This approach, together with new urban mobility and sustainability paradigms, seeks to ensure that cities, as they grow and develop, ensure equity in job opportunities, income, housing, access to essential services, and transport infrastructure [31–33]. These elements encourage sustainable mobility, which has been conceptualised as a low-carbon travel model that seeks to improve people’s quality of life and reduce the negative effects of the car-based transport model [30]. It seeks to improve air quality indicators, congestion, and the proportion of public transport and non-motorised trips.

According to the report of the Economic Commission for Latin America and the Caribbean [34], the countries of the region should focus on urban planning and urban land policies under a logic of social inclusion, with investments in transport infrastructure that improve the conditions of public transport, benefit its use, contribute to the reduction of carbon dioxide emissions, and reduce inequalities.

However, in practice, how cities have expanded in both developed and developing countries has given rise to shades of inequity in the provision of goods and services that impact sustainable mobility, especially in Latin America. In developing countries, elements of the built environment have been shown to have a significant influence on people’s trips when socioeconomic factors, land use regulations, and the balance between employment and housing are taken into account [35].

The work of [36] analyses spatial inequality in urban areas of cities in developing countries such as Bolivia, Ethiopia, Jamaica, Ecuador, and Peru. It suggests that spatial inequality in developing countries is linked to the development over time of distinct urban areas and to the deprivation that undermines the benefits of physical proximity that urban residence can offer. It also suggests that policy-making processes must incorporate geographic and social components to achieve more strategic and equitable urban development.

A study on socio-spatial inequalities and urban transformation in Rome-Italy shows that the urban quality, reflecting the income and wealth of inhabitants, is significantly correlated with the centrality of their location, the accessibility to a wide range of municipal functions and services and the absence of elements of social and labour unrest. They highlight the importance of identifying local policies capable of reducing local inequalities and promoting environmental sustainability and resilience [37].

Recent work reports empirical evidence on the relationship between social and spatial inequalities and highlights the fact that public policies should aim to mitigate socioeconomic and urban disparities to reduce spatial inequalities, especially in the provision of services to disadvantaged groups [38, 39].

In examining how developed countries have overcome the problems of inequity in urban areas to make way for sustainable mobility, it is observed that international experience shows that to achieve cities where public transport and non-motorised travel are at the centre of mobility, cities must ensure economic diversification [40–44], ensure the equitable distribution of urban amenities [45]; provide an efficient and responsive multimodal transport system that meets the mobility needs of cities [45], also ensure the equitable distribution of land uses in the different locations of the city [11, 12, 45, 46].

Pro-public transport cities, which focus on improving local accessibility through pedestrian-scale neighbourhood planning, are concerned with controlling urban sprawl and promoting gentrification as a mechanism to reduce long commuting distances [23, 47–49]. These characteristics contrast with pro-private vehicle characteristics that promote urban sprawl, favour recreational urban infrastructure such as highways [50, 51], and conceive of private vehicle ownership and use as a symbol of wealth and prosperity [48].

Leading cities in sustainable mobility include Barcelona, Portland and Melbourne [21, 52, 53]. However, in developing countries such as those in Latin America, given the current level of urban development, the inequitable spatial distribution of goods and services and the need for robust multimodal transport systems remain the challenges to overcome [54]. Consequently, the mobility challenges and the coordination of transport and land use in developing countries differ substantially from those in more prosperous and advanced countries [31, 54].

In the context of Latin America and, in particular, Chile, urban spatial inequity is a persistent element and has multiple effects on urban dynamics and behaviour patterns [55, 56]. In the context of Chile, since the last decade, there has been progress in investments to improve urban equipment and transport infrastructure, mainly in the city of Santiago de Chile. These investments have meant progress in the multimodal transport system and improvements in the urban equipment of the city. However, as a result of the segregation of the city [57–59] and spatial inequities in urban amenities [60], the most vulnerable segments of the population, located in the poorest communes, continue to experience urban inequalities and the negative externalities of the absence of mixed land use and transport management that are reflected in the travel behaviour of public transport users.

To our knowledge, in the international literature, there are no studies that evaluate the impact inequity of urban facilities, mixed land use, and urban amenities on trips completed in sustainable mode (trips by public transport and by non-motorised modes), considering the different dimensions of travel (subsistence, maintenance, and occasional trips), that reflect the main motivations for travel in a city. Consequently, in this paper, we fill this gap taking the city of Santiago de Chile as a case study.

We use a Poisson regression model and its geographical extension, the geographically weighted Poisson regression model (GWPR). This model allows us to recognise the heterogeneous behaviour of the data in space. For this purpose, we estimate two models: a regression for the entire sample and a regression through spatial regimes, where we divided the sample by communes. Our methodological approach is easy to apply in other contexts and cities, thanks to the use of freely available data such as Google Maps and Open Street Maps. Moreover, using this type of information allows for monitoring and policy design over the years.

We provide inputs to the empirical evidence related to developing countries such as Chile, which, due to the characteristics of the country, the results can be used as lessons for the rest of the developing countries. In addition, a particularity of this study is to analyse travel based on three dimensions (subsistence, maintenance, and occasional trips). This disaggregated analysis allows us to have a better understanding of the phenomenon and allows us to see disaggregated effects for each dimension of travel.

The rest of the article is organised as follows: First, it presents the main modelling approach and the structure of the models. Subsequently, it introduces the case study, describes the data, and describes the variables used. Then, it presents the results and discussion. Finally, we present the conclusions, policy implications, and limitations.

## Material and method

In this section, we present the procedures used in the development of this research and provide the theoretical justification for the use of the models employed. First, the entropy index is presented, and the rationale for using it as a proxy for measuring mixed land use is justified based on the literature.

We then present and provide the theoretical basis of the functional model used and the functional forms of the specifications used in the econometric estimations. Finally, the section concludes by indicating the advantages of the modelling and estimation approach used.

### Entropy index

According to [11, 61–63], we measure land use by entropy index. Six land uses, classified as residential, industrial, commercial, state, open space green areas, and other uses, were considered in calculating the entropy index, which is defined in Eq 1.


$$EI_{z}=-\sum_{i=1}^{k}\frac{p_{i^{*}}\text{ln}\;\left(p_{i}\right)}{\text{ln}\;(k)}$$ (1)

$$0\leq {EI}_{z}\leq 1$$

Where

*EI*_*z*_: Entropy index of the area

*p*_*i*_: Share of a land use type.

*k*: This is the number of land use categories included in the index calculation.

The calculated values of the entropy index are used as input in the estimation of the Poisson regression model and its spatial variant, the geographically weighted Poisson regression model (GWPR). These models are used to determine the impact of land use and urban attributes on the trip number in sustainable transport modes (public transport trips and non-motorised trips), as done in the work of [64–66]. The theoretical elements justifying the use of the Poisson model and its spatial variant GWPR are given below. The different equations that define the models are presented, and an explanation of the components of each equation is given.

### Poisson regression model (PR) and Geographically Weighted Poisson Regression (GWPR)

From an analytical point of view, we use a functional model divided into two parts. The model (1) is defined as *Y* = *f* (*X*, *Z*) = *PR* (*X*, *Z*), where *Y* = number of trips in sustainable modes, *X* = land use and *Z* = urban attributes. Then, given that there is heterogeneity in space, we seek to make this relation (1) sensitive to location. Since it is hypothesised that the relation (1) changes according to where they are located (X and Z) in space due to their heterogeneity, thus (1b) is defined as

*Y*(*s*) = *f*(*X*(*s*),*Z*(*s*)) = *Local Spatial PR* (*X*, *Z*). Were *s in a pair* (*u*, *v*). This allows us to know how model (1) parameters change as "s" changes.

This approach can also deal with the problem of overdispersion (*μ* < *σ*^2^). In practice it may be that the variance and mean change by blocks within the territory by sub-areas. Including the location "s" adjusts the over variance, allowing the generation of models in territorial blocks.

However, this model (1b), Local Spatial PR, cannot be estimated (except perhaps in a Bayesian mode).

The substitute model is the GWPR, which only models *y*_*i*_(*s*) as a direct function of attributes and space (it does not do so as a Poisson directly as it is not a probability model), where the model acquires several weaknesses as opposed to the PR.

The positive elements of Poisson models are that they are typically suitable for discrete count data, allow easy interpretation of the impact of variables and are computationally efficient. It is also flexible to capture complex effects such as zero inflation and overdispersion or to capture random parameters when there is unobserved heterogeneity.

Unfortunately, model (1b) is not precisely a Poisson, and the literature recognises some weaknesses related to (a) the presence of overdispersion, (b) multicollinearity, (c) model selection, (d) statistical inference and (e) limited interpretability. In this model, there are ways to correct for overdispersion, see [67]. Multicollinearity occurs when spatial lags are included, which enlarges the errors and makes it difficult to interpret the individual coefficients. One way to resolve this is to use Ridge Regression-type regularisation techniques, which can introduce biases. Model selection is affected by sensitivity to the bandwidth parameter that determines the spatial weight of the observations. Since the model uses a local regression technique, bootstrapping is necessary for inference. Finally, the model has limited interpretability since, although it allows highlighting heterogeneity, it is difficult to interpret the role of the attributes in the relationship, which is partly solved with visualisation techniques. However, it is difficult to see the whole.

Even so, it is a widely used and accepted technique in the scientific community for modelling spatial heterogeneity. See, for example, [68–72]. The specifications of the analytical model 1 (Poisson Model) and 1b (Weighted Poisson Regression) are presented in the following sub-sections.

### Poisson model

The Poisson regression model is a type of regression analysis for modelling count data under the assumption that the explained variable follows a Poisson distribution. A crucial assumption in empirical modelling is that the mean and variance must be equal; although this assumption is the exception rather than the rule, our data set used validates the overdispersion test, which allows the estimation of Poisson regression models rather than negative binomial models.

The Poisson model considered pooled measures of urban amenities and disaggregated data on individuals’ trips. A separate model was estimated for the three trip dimensions analysed (subsistence trips, maintenance trips and discretionary trips). This approach was preferred to estimating a separate model for each travel dimension, as it allows a direct comparison of travel behaviour results through semi-elasticities that complement the analysis of the spatial effect captured in the geographically weighted regression model. The number of trips *y*_*i*_ by mode of transport on a working day *d* *ϵ* *D*, can be modelled by means of a Poisson regression model, defined as:

$$P\left(y_{i}=d/\mu_{i}\right)=\frac{\exp\left(-u_{i}\right)\cdot \mu_{i}^{d}}{d!}\;d=0,1\ldots ,D$$ (2)

The prediction rate, *μ*_*i*_, is both the mean and variance of *y*_*i*_ and is defined as follows:

$$\mu_{i}=E\left(y_{i}=y_{i}|x_{i}\right)=\exp\left(\beta^{\prime }x_{i}\right)$$ (3)

The estimation incorporates random parameters to account for unobserved heterogeneity. The prediction rate of the number of trips per mode is calculated as a function of the different generic and mode-specific explanatory variables and trip dimensions. Eq 4 calculates the elasticities.


$$\eta =\frac{\partial E\left(y_{i}|x_{i}\right)}{E\left(y_{i}|x_{i}\right)}*\frac{x_{i}}{\partial x_{i}}=\beta \cdot x_{i}$$ (4)

### Geographically Weighted Poisson Regression (GWPR) model

The GWPR allows each regression parameter to vary over the study area to capture the spatially varying data relationships. In this way, the model can capture the local variation between the number of trips an individual performs and some explanatory variables, including accessibility measures to basic amenities and socioeconomic characteristics. This model represents an improvement over traditional regression models, which assume uniform behaviour of variables across space. The GWPR captures the spatial behaviour of observations, allowing for greater flexibility when the phenomenon under study is suspected of exhibiting spatial heterogeneity, as in our case study. Thus, the GWPR enables a better understanding of how the relationship between the dependent and explanatory variables can vary across space. Finally, the GWPR reduces bias and improves the accuracy of estimates, enhancing the goodness-of-fit of the data [73]. Similar to [60], the estimated spatial model is determined by the following equation:

$$y_{i}=\beta_{0}\left(u_{i},v_{i}\right)+\sum_{k=1}^{K}\beta_{n}\left(u_{i},v_{i}\right)x_{ik}+\sum_{s=1}^{S}\beta_{s}\left(u_{i},v_{i}\right)x_{is}+\varepsilon_{i}$$ (5)

The dependent variable represents the number of trips that individuals make, and the independent variables are the urban attributes related to basic amenities and socioeconomic variables. We include *n* urban services to capture the level of access each individual has to urban amenities using the index proposed by [74]. Note that each parameter is a function of location (*u*_*i*_, *v*_*i*_) representing the spatial location of individuals. Thus, the model allows us to address the spatial heterogeneity that arises from the relationship under study. The parameters are estimated as follows:

$${\hat{\beta }}_{\left(u_{i},v_{i}\right)}={\left(X^{T}W\left(u_{i},v_{i}\right)X\right)}^{-1}X^{T}W\left(u_{i},v_{i}\right)y,$$ (6)

Where *W*(*u*_*i*_, *v*_*i*_) is a *n* by *n* spatial weight matrix, where off-diagonal elements are equal to zero, meanwhile diagonal elements are different to zero and denote the geographical weighting of each observation. To compute the W matrix that captures the relationship between observations and the regression points, the following bi-square function is defined:

$$W\left(u_{i},v_{i}\right)=\left\{\begin{array}{ll} {\left[1-{\left(\frac{d_{ij}}{b}\right)}^{2}\right]}^{2} & \;\text{if}\;d_{ij}<b\\ 0 & \text{Otherwise} \end{array}\right.$$ (7)

Where *d*_*ij*_ is the Euclidian distance, and the parameter b is the spatial bandwidth. This matrix varies across the location of observation *i* and allows closer observations to have more weight in the computation of model parameters than observations located farther away [75]. Also, an adaptative bi-square function allows the weighted scheme to vary across the space according to the data density. This approach allows for overcoming the issues associated with using a fixed kernel, especially in cases where data are less dense. With a fixed kernel, the standard errors of the coefficients can be high because the number of observations used is small, reducing the reliability of the estimated regression coefficients. This problem can be addressed through the estimation of GWPR with spatially varying kernels. Thus, under this approach, the bandwidth is large where the data is sparse, and where data is concentrated, the bandwidth is small.

### Case study, data sources, and variables used

#### The city

Santiago de Chile is the capital of the largest metropolitan region in Chile, with an area of approximately 640km^2^, as well as the city with the most significant number of inhabitants (6,310,000 inhabitants according to the 2017 population census), the city with the highest proportion of the private car fleet (39. 3%), according to data from the National Institute of Statistics INE-2017, and the only city in the country with a multimodal transport system and which also concentrates the most significant amount and diversity of economic activity in the country [11, 76–78]. Sustainable urban development and sustainable mobility play a key role in the objectives of the 2030 agenda for sustainable development in the new global and regional context of Latin America and the Caribbean [79]. Developed countries, pioneers in sustainable mobility, such as Norway, the Netherlands, Japan, South Korea, Germany, and England, have implemented a set of public policies aimed at stimulating the use of public transport, non-motorised travel, and the use of electric and hybrid vehicles, as a way to reduce CO_2_ emissions, concentrations of PM_2.5_ and O_3_ produced by the private use of the vehicle fleet. As in all countries in Latin America and Chile, progress in terms of sustainable mobility and the design of public policies aimed at strengthening sustainable mobility is still in its early stages. [21, 80, 81].

Each country has its own experience in implementing and progressing in terms of sustainable mobility; however, a common factor for all is that the promotion and adoption of sustainable mobility demands overcoming major economic, fiscal, and social challenges, as well as technical barriers associated with local technological development and urban infrastructure required for the massification of sustainable mobility [82].

The experience of countries pioneering sustainable mobility shows that public policies aimed at improving public transport, upgrading and building new transport infrastructure, and the provision of goods and services have the most significant impact on the advancement of sustainable mobility [54, 83, 84].

However, the effectiveness and success of different policies in these countries vary widely, depending on the economic and social context, differences in demographic characteristics, urbanisation developments, regional characteristics and individual travel patterns [85]. Intending to advance sustainable urban development and mobility implementation in Chile, the Chilean Government formulated the National Urban Development Policy and the National Strategy for Sustainable Mobility that seeks to position Chile as a reference in the region.

In the last decade, the city of Santiago has begun to encourage the use of public transport and the generation of non-motorised trips [86]. The State has made great efforts in the construction and adaptation of transport infrastructure such as New public transport corridors, an increase of bus stops and routes, construction and adaptation of bicycle routes, as well as the adequacy of pedestrian spaces (Management reports. Ministry of Transport and Telecommunications, Government of Chile); however, mobility reports from the Santiago de Chile origin-destination survey, show that between 2001 and 2012, the use of private vehicles increased by 5.1%, while the number of trips on foot and by public transport decreased by 10.1%.

In Chile, spatial inequality, the urban development of regions and municipalities, and the income levels of different population segments are quite unequal [87–89].

The city of Santiago is the largest example of these inequalities, reflecting spatial inequity in terms of the provision of urban amenities, the provision of goods and services, transport infrastructure and income distribution [58, 59, 77]. This fact makes urban conditions and household mobility patterns a relevant heterogeneous component in mobility, time and monetary transaction costs. These aspects make the city of Santiago an international benchmark case study, drawing valuable lessons to inform policy design in countries with similar urban and social characteristics.

### Data source

**Fig 1:**
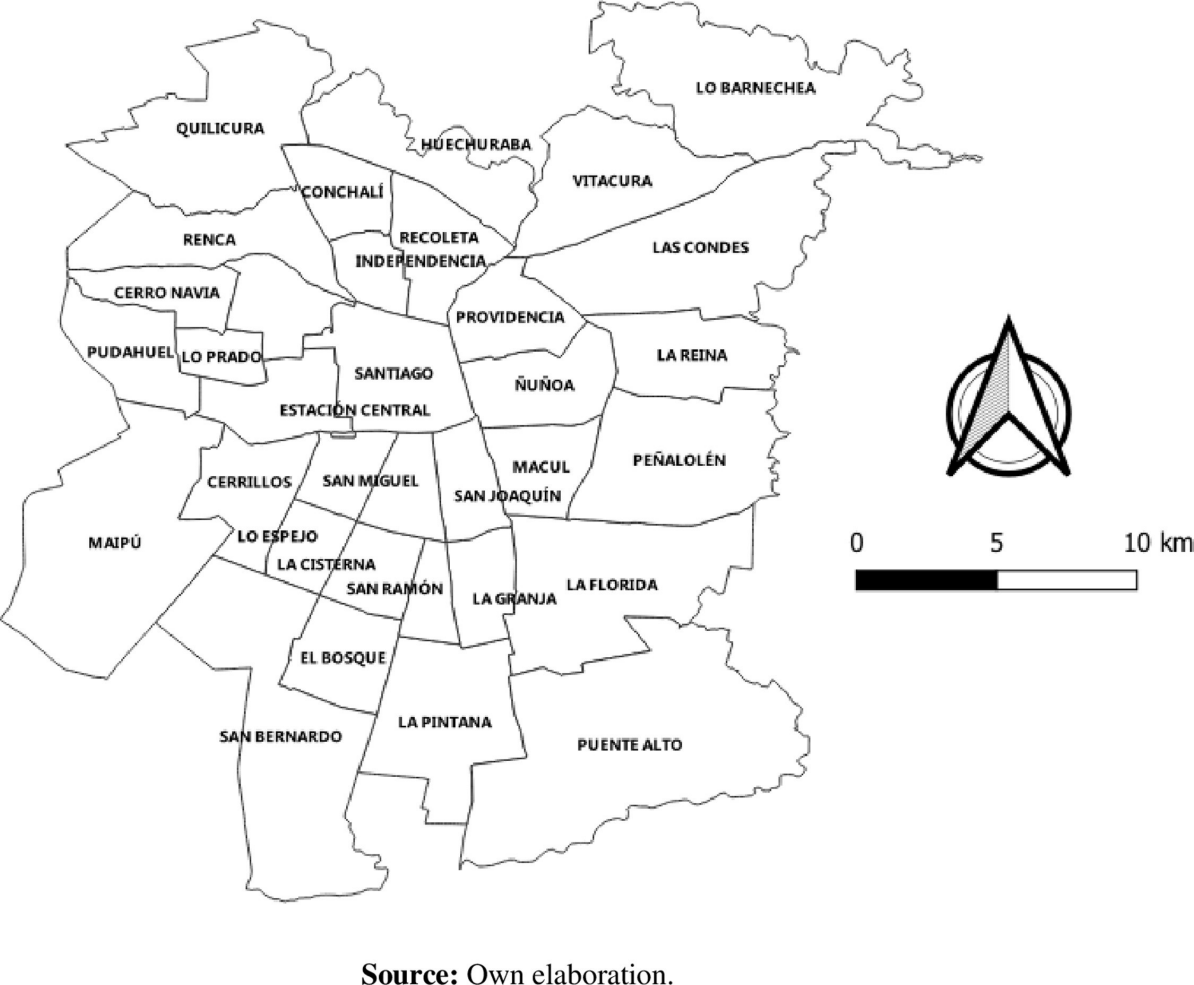
Municipalities of Santiago to be considered for analysis. The municipalities are autonomous corporations under public law, with legal personality and assets, whose purpose is to satisfy the needs of the local community and ensure its participation in the economic, social, and cultural progress of the respective communes (Law Nº 18.695 de 2006. Constitutional Organic Law of Municipalities. Government of Chile).

We use the 2012 Mobility Survey (EOD-2012), the 2017 Population and Housing Census of the National Institute of Statistics (INE-2017), and data on land use and urban equipment extracted from OpenStreetMap in 2020. The key information on Open Street Maps, such as the presence of urban facilities, was cross-checked with official sources such as the Ministry of Urban Planning and the National Institute of Statistics. The Santiago de Chile Origin-Destination Survey (EOD-2012) is the most recent mobility survey for the city of Santiago de Chile. It contains 96,013 trips from 40,889 users, representative at the municipal or commune level. For the purposes of the model estimations, we only consider 51,819 trips made on weekdays from 22,541 inhabitants surveyed for 34 municipalities or communes (Fig 1), which have coverage of the Metropolitan Mobility Network.

The variables used from the (EOD-2012) are income, motorisation rate, public transport trips, non-motorised trips (walking and cycling) and trips in private vehicles for subsistence (study and work trips), maintenance (shopping and personal care trips), and discretionary (use of leisure time) trip dimensions. The definition of these three dimensions of travel considers aspects of individuals’ lifestyles, households, and travel demand, in line with the work of [90, 91].

The population density variable was obtained from the 2017 Population and Housing Census of the National Statistics Institute. The land use and basic equipment variables associated with transport, education, health, commerce, and leisure activities were obtained from OpenStreetMap. Table 1 describes the variables.

**Table 1:** Variables description.

Variable	Source	Mean	SD
**Trip**			
Public transport (Daily trips)	Origin Destination Survey	3.5	1.2
Non-motorised (Daily trips)	Origin Destination Survey	2.6	0.5
Private Trips (Daily trips)	Origin Destination Survey	2.1	0.3
**Land Use**			
Commercial (km²)	Open Street Maps	0.57	0.77
Residential (km²)	Open Street Maps	14.99	40.49
Industrial (km²)	Open Street Maps	1.71	3.42
State (km²)	Open Street Maps	0.96	1.97
Open spaces and green areas (km²)	Open Street Maps	1.10	1.30
Other (km²)	Open Street Maps	10.13	18.39
EI (Entropy index, Rank of 0–1)	Open Street Maps	0,54	0,18
**Vehicle ownership**			
TM (Engine ownership rate In per cent)	Origin Destination Survey	0,76	0,7
**Road density by municipality**			
Primary roads	Open Street Maps	8,63	6,62
Secondary roads	Open Street Maps	7,39	5,04
Tertiary roads	Open Street Maps	7,58	4,16
Pedestrian streets	Open Street Maps	1,32	2,48
Cycleway	Open Street Maps	0,78	0,79
**Transport infrastructure**			
Number Bus stops per km²	Open Street Maps	24	12,73
Number Subway stations per km²	Open Street Maps	0,25	0,35
Number Fuel stations per km²	Open Street Maps	0,67	0,62
**Health Facilities**			
Number Parking per km²	Open Street Maps	0,87	1,22
Number Pharmacy per km²	Open Street Maps	1,68	2,07
Number Clinics per km²	Open Street Maps	0,19	0,24
Number Hospital per km²	Open Street Maps	0,17	0,17
Number Other health per km²	Open Street Maps	0,88	1,23
**Commercial (Number per km²)**			
Number Bank per km²	Open Street Maps	1,13	1,88
Number Restaurants per km²	Open Street Maps	4,97	8,28
Number Supermarket per km²	Open Street Maps	0,7	0,48
**Educational establishments**			
School per km²	Open Street Maps	3,95	2,45
Number kindergarten per km²	Open Street Maps	1,92	0,9
**Open spaces and green**			
Park (km²)	Open Street Maps	3,79	3,27
**Socioeconomic Variables (in thousands)**	Open Street Maps		
Population density (habitants per km²)	(INE,2017)	7447	4118

*The descriptive statistics represent the averages calculated for the municipalities considered in the study.

### Variables

Based on the literature discussed in the introduction section, in particular the work of [6, 21, 41, 43, 44, 63, 71, 92, 93], we consider a set of variables that measure the mixed land use and the urban amenities required for the development of the main activities of daily life, such as study, work, leisure, shopping, personal errands, etc. These activities are grouped into the dimensions of subsistence (study, work), maintenance (shopping, medical appointments, personal errands, etc.), and leisure.

The set of urban variables considered in the analysis was: (a) Transport infrastructure (Density of available bus stops, density of metro stations, density of fuel stations, density of available parking, density of primary, secondary, tertiary, pedestrian, and cycle routes), (b) Health infrastructure (density of pharmacies, clinics, other health centres), (c) Commercial infrastructure (density of banks and restaurants), (d) Educational infrastructure (density of schools and kindergartens), (e) Leisure and free time (parks and green areas).

## Results and discussion

Table 2 shows the results of the Poisson model estimations for subsistence trips (educational and work trips). The results show that the entropy index, tertiary road density, metro station density, kindergarten density, and population density positively impact the number of trips generated by public transport.

**Table 2:** Parameters of the Poisson regression model for subsistence trip.

Public Trips		Private Trips		Non-motorised Trips	
Variable	Value	Variable	Value	Variable	Value
Constant	0.850(2.3) ***	Constant	1.326 (1.8) **	Constant	0.771(10) ***
EI	0.016(3.69) ***	EI	-0.227(-2.5) ***	EI	0.4368(3.6) ***
Primary	-	TM	0.214(4.8) ***	Tertiary	0.014(2.2) ***
Secondary	-	Primary	0.035(5.48) ***	Pedestrian	-0.014(-1.5) *
Tertiary	0.008(3.50) ***	Secondary	0.016(2.9) ***	Cycleway	0.139(4.6) ***
Bus stop	-0.082(-3.5) ***	Tertiary	-0.014(-2.2) ***	School	0.030(1.9) **
Subway stations	0.301(7.3) ***	Fuel stations	-	Kindergarten	-0.129(-2.6) ***
School	0.026(1.8) **	Parking	0.045(2.8) ***	Ln (Density Population)	-1.E-09(-1.2)
Kindergarten	0.076(2.1) ***	School	-0.321(-8.1) ***	Ln (Income)	0.0008(1.5)
Ln (Density Population)	-3.E-05(-1.64)**	Kindergarten	0.031 (4) ***		
Ln (Income)	0.03(1.7) ^**^	Ln (Density Population)	-1.E-05(-1.2)		
		Ln (Income)	0.008(3.46) ^***^		
N	4130	2152		1029	
Log-likelihood	-6521.6742	-4066.5572		-1861.3784	
Deviance goodness-of-fit	1756.515	1743.693		715.8861	
Prob > chi2	1	1		1	
Pearson goodness-of-fi	2484.070	2047.392		841.1836	
Prob > chi2	1	0.9253		1	
AIC	13061.35	8155.114		3736.757	

Signif 0.05*** 0.1** 0.2*

Variables such as primary and secondary road density are not significant. On the other hand, the density of bus stops, as well as the density of schools, have a negative impact on the number of public transport trips. The elasticities of the estimated model are shown in Table 3. The results show that controlling for the other variables, for every 1% increase in the value of the entropy index measuring land-use diversity, the number of public transport trips increases by 8.2%.

**Table 3:** Elasticity for subsistence trips.

Elasticity in Public Trips (*η*)		Elasticity in Private Trips (*η*)		Elasticity in Non-motorized(*η*)	
Variable	%	Variable	%	Variable	%
EI	8.2%	EI	-24.4%	EI	20.7%
Primary	-	TM	19.3%	Tertiary	4.3%
Secondary	-	Primary	3.6%	Pedestrian	1%
Tertiary	1.6%	Secondary	1.60%	Cycleway	14.9%
Bus stop	-0.8%	Tertiary	-1.40%	School	3%
Subway stations	35.2%	Fuel stations	-	Kindergarten	-13.8%
School	2.70%	Parking	3.1%		
Kindergarten	7.9%	School	-4.6%		
		kindergarten	27.5%		

Controlling for the other variables, it is also observed that a 1% increase in the number of metro stations per square kilometre increases the number of public transport trips by 35.2%. In the case of non-motorised trips, controlling for the other variables, for every 1% increase in the value of the entropy index measuring land-use diversity, the number of non-motorised trips increases by 20.5%.

Table 4 shows the estimates for maintenance trips for the three modes considered. The results show that the entropy index positively impacts the number of maintenance trips completed by public transport and non-motorised trips and negatively impacts the number of maintenance trips completed by private vehicles.

**Table 4:** Parameters of the Poisson regression model for maintenance trips.

Public Trips		Private Trips		Non-motorised Trips	
Variable	Value	Variable	Value	Variable	Value
Constant	1.286 (20) ***	Constant	1.441(19) ***	Constant	1.027(19.36) ***
EI	0.623(5.5) ***	EI	- 0.480(-3.59) ***	EI	0.374(3.52) ***
Primary	0.037(5.4) ***	TM	0.062(1.73) **	Tertiary	0.012(2.1) ***
Secondary	-	Primary	0.020(2.16) ***	Pedestrian	0.010(1.89) **
Tertiary	-	Secondary	-	Cycleway	0.130(3.61) ***
Bus stop	-	Tertiary	0.025(2.97) ***	Pharmacy	0.091(2.17) ***
Subway stations	0.195(-2.4) ***	Fuel stations	-	Clinics	-
Supermarket	-0.486(6.0) ***	Parking	0.064(1.73) **	Hospital	0.669(3.45) ***
Pharmacy	-0.325(-5.0) ***	Supermarket	0.177(2.52) ***	Other/Health	0.042(1.79) **
Clinics	1.086(5.1) ***	Pharmacy	-	Bank	0.038(1.48) *
Hospital	-	Clinics	-	Supermarket	-
Other/Health	0.103(5.16) ***	Hospital	0.409(1.62) *	Ln (Density Population)	-
Bank	0.194(4.6) ***	Other/Health	0.121(3.29) ***	Ln (Income)	-
Ln (Density Population)	2.E-05(2.7) ***	Bank	-	-	-
Ln (Income)	-	Density Population	2. E-05(1.85) **	-	-
		Ln (Income	-		
N	1300	1504		1495	
Log-likelihood	-22077.068	-3348.2488		-3025.9785	
Deviance goodness-of-fit	696.157	1614.397		1496.891	
Prob > chi2	1	0.60463		0.6020	
Pearson goodness-of-fi	852.3	16.81145		1763.745	
Prob > chi2	1	0.6312		0.8254	
AIC	4433.414	6716.498		6058.298	

Signif 0.5*** 0.1** 0.2*

It is also observed that the density of primary tracks positively impacts the number of maintenance trips completed in all three modes of travel. The number of clinics and hospitals per square kilometre also positively impacts the number of trips completed in all three modes. This result makes sense and can be explained by the diversity of types of medical consultations or emergencies, age range, and life cycle of individuals.

The results also indicate that the number of supermarkets per square kilometre positively impacts the number of trips completed by private transport and negatively impacts the number of trips completed by public transport. One explanation for this result may be that people prefer to use a private vehicle for larger purchases because of the convenience offered by the private vehicle, as shown in the work of [94].

The results in Table 5 suggest that controlling for the other variables, for every 1% increase in the value of the entropy index measuring land-use diversity, the number of non-motorised trips increases by 25.7%. This result may be explained by the fact that a greater land use diversity ensures a more significant provision of goods and services close to the residence, reducing travel times.

**Table 5:** Elasticity for maintenance trips.

Elasticity in Public Trips (*η*)		Elasticity in Private Trips (*η*)		Elasticity in Non-motorized(*η*)	
Variable	%	Variable	%	Variable	%
EI	16.4%	EI	-21.7%	EI	25.7%
Primary	3.8%	TM	6%	Tertiary	1%
Secondary	-	Primary	1.9%	Pedestrian	1%
Tertiary	-	Secondary	-	Cycleway	14%
Bus stop	-	Tertiary	2.5%	Pharmacy	9%
Subway stations	17.7%	Fuel stations	-	Clinics	-
Supermarket	-28.5%	Parking	6.6%	Hospital	35%
Pharmacy	-27.7%	Supermarket	16.3%	Other/Health	4%
Clinics	12.3%	Pharmacy	-	Bank	4%
Hospital	-	Clinics	-	Supermarket	-
Other/Health	10.8%	Hospital	18.6%	-	-
Bank	21.4%	Other/Health	13%	-	-
	-	Bank	-		

Tables 6 and 7 present the results for discretionary trips (leisure trips) for the three modes of transport. It is observed that mixed land use has a positive impact on the number of trips completed by public transport, but not on the number of trips completed by private vehicles and non-motorised trips.

**Table 6:** Parameters of the Poisson regression model. Discretionary trips.

Public Trips		Private Trips		Non-motorised Trips	
Variable	Value	Variable	Value	Variable	Value
Constant	0.541(3.2)***	Constant	1.254(7.8) ***	Constant	1.320(9.9) ***
EI	0.761(3.0) ***	EI	-	EI	-
Primary	0.025(2.0) ***	TM	0.232(2.5) ***	Pedestrian	0.016(1.55) **
Secondary	-	Primary	0.031(13.7) ***	Tertiary	0.049(2.02) ***
Tertiary	-	Secondary	0.029(14.9) ***	Cycleway	0.118(1.8)**
Bus stop	0.006(1.3)**	Tertiary	0.017(7.7) ***	Restaurant	0.044(1.9)**
Subway stations	-	Fuel stations	0.249(12.1) ***	Park	0.022(2.96) ***
Park	-	Parking	0.030(2.7) ***	Ln (Density Population)	-2.E-05(-2.04)***
Restaurant	-0.011(-1.4)**	Restaurant	0.014(10.7) ***	Ln (Income)	-
Ln (Density Population)	2.E-07(-1.6)**	Park	0.021(6.1) ***	-	-
Ln (Income)	-	Ln (Density Population)	6.E-05(-17.9) ***		
		Ln (Income)	0.041(7.7) ***		
N	254		329	398	
Log-likelihood	-453.7711	-718.2100		-710.1413	
Deviance goodness-of-fit	154.352	3758364		229.6938	
Prob > chi2	1	0.0128		1	
Pearson goodness-of-fi	174.8833	4092794		262.9392	
Prob > chi2	0.9997	0.5004		1	
AIC	929.5423	1458.177		1436.283	

Signif 0.5*** 0.1** 0.2*

**Table 7:** Elasticity for discretionary trips.

Elasticity in Public Trips (*η*)		Elasticity in Private Trips (*η*)		Elasticity in Non-motorized(*η*)	
Variable	%	Variable	%	Variable	%
EI	18%	EI	-	EI	-
Primary	3%	TM	26.1%	Pedestrian	1.6%
Secondary	-	Primary	3.1%	Tertiary	4.8%
Tertiary	-	Secondary	3%	Cycleway	12.6%
Bus stop	0.6%	Tertiary	1.7%	Restaurant	4.3%
Subway stations	-	Fuel stations	22%	Park	2.2%
Park	-	Parking	3.1%		
Restaurant	2%	Restaurant	1.4%		
-	-	Park	2.1%		

The calculation of elasticities in Table 8 shows that, when controlling for the other variables, a 1% increase in the land use mix measure increases the number of public transport trips by 18%. An important result concerning sustainable mode travel is the elasticity value of the increase in the length of cycleways. Controlling for the other variables, a 1% increase in cycleway coverage per square kilometre increases the number of leisure trips completed by bicycle by 12.6%.

**Table 8:** GWPR model.

Dependent variable (Trips)	Poisson			Geographically Poisson Weighted Regression Results				
Co-variables	Est.	t(Est/SE)	p-value	Mean	STD	Min	Median	Max
***Socioeconomic***								
Gender	-0,0690	-7,1740	0,0000	-0,065072	0,021507	-0,100188	-0,067668	-0,006757
Income	0,0000	3,9050	0,0000	0,000000	0,000000	0,000000	0,000000	0,000000
Basic education	-0,0690	-7,1740	0,0000	0,136167	0,115180	-0,207822	0,163628	0,355895
Secondary education	0,0730	0,6300	0,5280	0,138477	0,146103	-0,148599	0,113159	0,439626
Technical education	0,1000	0,7670	0,4430	0,108037	0,153238	-0,248956	0,156279	0,384306
Vocational education	0,0550	0,3440	0,7310	0,062269	0,105312	-0,198862	0,054343	0,261036
Postgraduate studies	-0,0090	-0,0890	0,9290	0,086136	0,117862	-0,205764	0,094652	0,279208
Master’s studies	0,0050	0,0490	0,9610	0,075831	0,112321	-0,228147	0,111048	0,245205
Doctoral studies	0,0130	0,1210	0,9040	0,138234	0,106530	-0,146122	0,128769	0,318737
**Urban attributes**								
Municipal secondary schools (Radius of coverage 3 km)	0,0000	-1,1560	0,2480	-0,000008	0,000017	-0,000047	-0,000010	0,000025
Private secondary schools (Radius of coverage 3 km)	0,0000	7,4840	0,0000	0,000002	0,000007	-0,000019	0,000001	0,000025
Subsidised private secondary schools (Radius of coverage 3 km)	0,0000	-1,3590	0,1740	0,000001	0,000003	-0,000011	0,000001	0,000006
Kindergartens (Radius of coverage 3 km)	0,0000	6,0490	0,0000	0,000009	0,000007	-0,000004	0,000007	0,000025
Sports equipment (Radius of coverage 4 km)	0,0000	-2,0480	0,0410	-0,000003	0,000003	-0,000010	-0,000003	0,000005
Parks (Radius of coverage 5 km)	0,0000	1,0510	0,2930	-0,000005	0,000011	-0,000030	-0,000005	0,000022
Articulated bus stops (Radius of coverage 1 km)	0,0000	-1,3920	0,1640	0,000001	0,000002	-0,000004	0,000001	0,000006
Family Health Centres (Radius of coverage 4 km)	0,0000	4,8730	0,0000	0,000022	0,000018	-0,000045	0,000028	0,000056
Clinics (Radius of coverage 5 km)	0,0000	-0,7680	0,4430	-0,000006	0,000014	-0,000027	-0,000010	0,000042
Pharmacies (Radius of coverage 4 km)	0,0000	-3,4950	0,0000	-0,000002	0,000008	-0,000038	-0,000001	0,000011
Restaurants (Radius of coverage 5 km)	0,0000	0,9370	0,3490	-0,000001	0,000003	-0,000009	-0,000001	0,000004
Fast food outlets (Radius of coverage 5 km)	0,0000	0,7410	0,4590	0,000003	0,000006	-0,000005	0,000002	0,000040
Banks (Radius of coverage 5 km)	0,0000	3,3320	0,0010	0,000009	0,000011	-0,000006	0,000005	0,000066
Intercept	0,9170	8,7510	0,0000	0,923827	0,158695	0,595021	0,909702	1,284200
**Observations**	17009			**Observations**	17009			
AIC	56893,95			AIC	8709,477			
AICc	8993,925			AICc	8711,289			

Note: To determine the relevance of the GWPR model, we verified the existence of spatial heterogeneity in the residuals through the Chow Test. The null hypothesis indicates that coefficients estimated from regression for the entire sample and coefficients estimated through spatial regimes are equal. For the regime regression, we divided the sample by communes. The result of this contrast is 70.198 (28;16,953), which is highly significant. This result suggests that the regression coefficients are not constant across regimes, indicating the existence of spatial heterogeneity.

**Fig 2:**
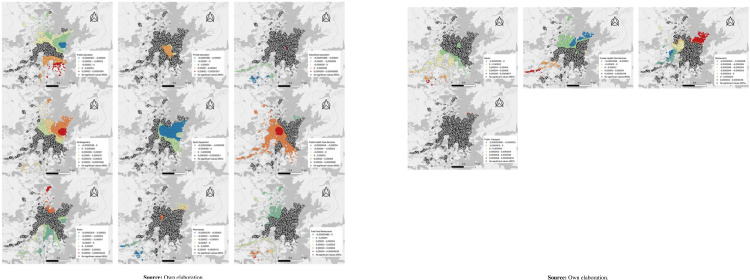
Spatial distribution of GWPR model coefficients. The colors reflect how the urban amenities included in the GWPR are distributed and concentrated in the space.

Table 8 presents the geographically weighted Poisson regression (GWPR) model coefficients. The geographical system used in the model estimation was WGS84. Fig 2 illustrates the coefficient spatial variation for accessibility variables. The results show that the spatial distribution of demographic variables, primary and secondary schools, and transport infrastructure are good travel predictors, but the spatial distribution of attributes such as banks, clinics, and parks is not.

The spatial variation of the estimated coefficients illustrated in Fig 2 shows the existence of the phenomenon of spatial heterogeneity. This implies high spatial inequality of urban attributes among the city’s communes and diversity of demographic characteristics of people in each commune.

The presence of spatial heterogeneity revealed by the GWPR Model reflects the inequity and persistent urban segregation in the city of Santiago since the 1990s, as shown in the work of [95]. In practice, this phenomenon translates into the impossibility for the city’s inhabitants to have access to a variety of facilities and to the provision of quality goods and services to satisfy their needs.

In the context of Santiago de Chile, these differences deepen spatial segregation but also have differential impacts on travel patterns, especially sustainable mode travel.

As shown in the work of [93], in the context of Santiago de Chile, the set of urban amenities and socio-demographic variables are a good predictors of travel mode choice. This fact makes the social dimension a relevant factor in designing and implementing urban development policies aimed at sustainable mobility. Given that urban sustainability presupposes reducing the prevailing patterns of inequality in the metropolitan region of Santiago.

Local authorities should take advantage of the marked differences produced by the persistence of spatial inequity in urban facilities in terms of sustainable travel patterns to guide the design of public policies towards socio-urban integration and strengthen the residential integration or gentrification processes the city has experienced.

In this way, sustainable travel, especially public transport, can be favoured, as has happened in the case of developed countries such as Canada [96] and developing countries such as India [97].

However, the balance between the different types of urban and transport equipment must be taken care of since, for example, the fact that the density of bus stops has a negative impact on the number of expected trips by public transport may indicate that, although a greater presence of bus stops per square kilometre represents a key accessibility factor, it may also become a negative element for users, as a greater number of bus stops increases travel times and stopping times, and passenger boarding and alighting times. Population density has an almost null and negative effect; this result aligns with those reported in works such as [92, 98].

In the context of Chile, our results are consistent with the findings of [55, 99–101], who show that spatial inequity has persisted for more than three decades.

In the Latin American context, our findings are consistent with the work of [102, 103] for the metropolitan area of Mexico and the work [104] for the city of Bogotá. The findings of [102] indicate that accessibility to work plays a determinant role in commuting and that the poorest populations experience the most extended travel times. Due to the long distances to work, it is impossible to travel in non-motorised modes. The findings of [103] indicate that private enterprises related to the provision of everyday consumer goods and services have a better spatial distribution in high-income areas than in low-income areas. They also find that the supply of local employment in high-income areas is higher than that of all private enterprises in low-income areas. Including those in the health sector. [104] report that in the city of Bogotá, the concentration of public services in space causes functional segregation and limits urban mobility conditions.

Compared with some international work, our results align with the work of [105], who suggests that the planning of urban spaces should consider accessibility and infrastructure provision. These aspects highlight the importance of urban facilities for mobility and sustainable travel, as subscribed by [106], who show that the presence of new cycleways increases interest in switching from private transport to cycling.

## Conclusions, policy implications and limitations

In this paper, we analyse the impact of spatial heterogeneity and urban attributes on sustainable modes of travel. To do so, we use the city of Santiago de Chile as a case study. We use data extracted from Open Street Maps and Google Maps to estimate a geographically weighted regression model and a Poisson probability model.

The findings suggest an imbalance between the diversity of land use in the communes of the metropolitan region and the inequality in the number of urban facilities in the communes or municipalities considered in the study.

On the other hand, the analysis of trips across the three dimensions allowed for a more precise identification of the differentiated impacts of land use and urban facilities on trip generation by modal split.

In particular, the results indicate that about 28.1% of the communes have an entropy index greater than or equal to 0.7. This entropy index means these communes present an almost uniform land use distribution for the six land categories considered in the study (Commercial, Residential, Industrial, State, Open spaces and green areas, Other).

On the other hand, the results on land use indicate that 48.8% of the communes present an uneven distribution of mixed land use under the analysis of the six categories. This is because their entropy value is at most 0.5. The soil effect can be considered one of the factors causing spatial heterogeneity.

The results of the weighted regression model show that the spatial distribution of variables related to demographics, transport infrastructure, as well as primary and secondary schools are good predictors of travel behaviour.

The results of the Poisson model suggest that mixed land use has a positive impact on public transport trips. In particular, it is observed that a 1% increase in the value of the entropy index (a measure of land use diversity) increases the number of public transport trips by 8.2%.

Controlling for the other variables, it is also observed that a 1% increase in the number of metro stations per square kilometre increases the number of public transport trips by 35.2%.

The same analysis for private transport shows that a 1% decrease in the land mix indicator reduces subsistence trip generation by 21%. However, it is not significant for maintenance and leisure trips.

The results also suggest that, for maintenance trips, the transport infrastructure variables do not positively impact trips in all three modes (non-motorised, public transport, and private transport). For example, in the case of public transport, the presence of metro stations per square kilometre and supermarkets does not positively impact the generation of maintenance trips in this mode.

Some public policy recommendations based on the results derived from the study can be oriented to:

1. Urban planning policy should consider increasing the provision and supply of goods and services in areas with the most significant lack of them, providing more bicycle lanes, improving pedestrian lanes and increasing available green areas, prioritising communes with lower per capita income and higher population density, given that in these communes the number of subsistence and maintenance trips is proportional to population density.
  This would improve physical and actual accessibility to different goods and services, contribute to the reduction of travel times and encourage non-motorised transport.
2. Promote the culture of micro-neighbourhoods, taking advantage of the polycentric structure of the city so that the majority of maintenance and subsistence trips are generated and attracted within the same urban units [21]. This is one of the recommendations derived from experiences in Canada [107].
  The fact that accessibility to schools negatively impacts trips completed by public transport, we believe that by improving the supply of schools close to the place of residence, not only public transport trips will benefit, but also walking and cycling trips, as a higher concentration of schools close to residences shortens distances and thus travel times.
3. Increase educational campaigns to encourage cycling for journeys of less than 3 kilometres and repeatedly encourage citizens to make better use of the cycleway network, as suggested by [80].
4. Encourage cultural incentives that trigger structural changes in lifestyles and use of sustainable modes in the young school-age population, as well as in young employees. This would increase the proportion of people using sustainable modes of transport, as suggested by [21].

A limitation of this work is the lack of temporal analysis of land use and travel variation. The impossibility of having data containing the temporal dimension did not allow us to carry out this type of analysis.

It is also important to highlight the theoretical and empirical limitations inherent in the model used, as outlined in the methodologies section.

An extension to this work could involve the temporal dimension through other sources of information, such as the reconstruction and digitisation of city maps in different time periods.
